# High *P4HA1* expression is an independent prognostic factor for poor overall survival and recurrent‐free survival in head and neck squamous cell carcinoma

**DOI:** 10.1002/jcla.23107

**Published:** 2019-11-29

**Authors:** Qun Li, Zhisen Shen, Zhenhua Wu, Yi Shen, Hongxia Deng, Chongchang Zhou, Huigao Liu

**Affiliations:** ^1^ Department of Otorhinolaryngology Head and Neck Surgery Ningbo Medical Center Lihuili Hospital Ningbo Zhejiang China; ^2^ Department of Otorhinolaryngology Head and Neck Surgery Lihuili Hospital affiliated to Ningbo University Ningbo Zhejiang China; ^3^ Department of Otorhinolaryngology Head and Neck Surgery Ningbo Medical Center Lihuili Eastern Hospital Ningbo Zhejiang China; ^4^ Department of Otorhinolaryngology Head and Neck Surgery Ningbo Zhenhai Longsai Hospital Ningbo Zhejiang China

**Keywords:** bioinformatics, carcinogenesis, diagnosis, head and neck squamous cell carcinoma, *P4HA1*, prognosis

## Abstract

**Background:**

Prolyl 4‐hydroxylase subunit alpha 1 (*P4HA1*) plays a critical role in modulating the extracellular matrix and promoting tumor progression in various cancers. However, the association between *P4HA1* and head and neck squamous cell carcinomas (HNSCC) has not been thoroughly elucidated to date.

**Methods:**

*P4HA1* mRNA and protein expression in cancer and normal tissues were analyzed using The Cancer Genome Atlas (TCGA), Gene Expression Omnibus, and Human Protein Atlas databases. Quantitative PCR was applied to determine *P4HA1* mRNA expression levels in 162 paired HNSCC and adjacent normal tissues. The cBioPortal for Cancer Genomics was utilized to explore *P4HA1* genetic alterations in HNSCC. Then, KEGG analysis of *P4HA1* co‐expressed genes in HNSCC was conducted using ClueGo in Cytoscape.

**Results:**

*P4HA1* mRNA and protein levels were significantly increased in HNSCC tissues compared with normal tissues. High *P4HA1* expression in HNSCC tissues was significantly associated with tumor category, lymphatic metastasis and pathological stage. The area under summary receiver operating characteristic curve of TCGA and validation cohort was 0.887 and 0.883, respectively. Moreover, elevated *P4HA1* expression was associated with unfavorable OS (HR: 1.728, *P* = .001) and RFS (HR: 2.025, *P* = .002) in HNSCC patients.

**Conclusions:**

This integrated analysis provides strong evidence that increasing *P4HA1* expression is significantly associated with the carcinogenesis of HNSCC. Additionally, high *P4HA1* expression serves as both diagnostic biomarker and independent prognostic factor for poor OS and RFS in HNSCC patients.

## 1 INTRODUCTION

Head and neck cancers represent the sixth most common cancer worldwide. The vast majority (greater than 90%) are head and neck squamous cell carcinomas (HNSCC), such that the term head and neck cancer refers to cancer arising from the epithelium lining the of the upper aerodigestive tract (lip, oral cavity, pharynx, and larynx) and exhibiting microscopic evidence of squamous differentiation.^1^ According to the latest report of the International Agency for Research on Cancer, approximately one million new HNSCC patients were estimated to be clinically diagnosed in 2018 with greater than 542 943 deaths worldwide.^2^ The risk for developing HNSCC is associated with several traditional etiological factors, including cigarette smoking and alcohol abuse.^3^ Increasing evidence also demonstrates that infection with a high‐risk human papillomavirus (HPV) strain is associated with HNSCC and is an important favorable prognostic factor, especially for oral cavity and oropharynx cancer.^4^, ^5^ Although the recent diagnostic and therapeutic strategies have yielded some significant improvements, the 5‐year survival rate for HNSCC patients over the last decade remained at approximately 50%.^6^ Although multiple molecular mechanisms are associated with HNSCC initiation, growth, invasion, and metastasis, the exact pathogenesis of tumorigenesis remains unclear. Development of new technologies, such as microarray technology and next‐generation sequencing, has allowed for collection of large amounts of data to explore the key genes in the pathogenesis of HNSCC,^7^, ^8^ such as *CDKN2A*,^9^ *CDH1*,^10^ and *EGFR*.^11^ Therefore, the identification of oncogenic drivers and potential therapeutic targets is crucial for both early diagnosis and effective treatment for HNSCC.

Tumor hypoxia is an essential characteristic of the neoplastic microenvironment that may be correlated with cell proliferation, apoptosis, differentiation, vascularization/angiogenesis, genetic instability, tumor metabolism, tumor immune responses, and invasion and metastasis.^12^ Under hypoxic microenvironments, hypoxia inducible factor‐1 (HIF‐1) promotes extracellular matrix (ECM) remodeling by inducing prolyl 4‐hydroxylase subunit alpha 1 (*P4HA1*), prolyl 4‐hydroxylase subunit alpha 2 (*P4HA2*), and procollagen‐lysine, 2‐oxoglutarate 5‐dioxygenase 2 (*PLOD2*) expression, leading to changes in cancer cell morphology, adhesion and motility that enhance invasion and metastasis.^13^ Located at 10q22.1, *P4HA1* encodes an active catalytic subunit of prolyl 4‐hydroxylase that catalyzes the formation of 4‐hydroxyproline in collagen, which is essential to the formation and stabilization of the triple helical domain of newly synthesized procollagen chains.^14^ *P4HA1* was identified as hypoxia‐responsive gene and plays a critical role in regulating collagen biosynthesis.^15^ Previous evidence suggested that *P4HA1* overexpression plays a critical role in cancer progression. Hu et al^16^ reported that high *P4HA1* expression is correlated with the malignancy of gliomas and could serve as a prognostic indicator for patients with high‐grade gliomas. In human breast cancer, *P4HA1* plays an essential role in enhancing invasion and metastasis and is significantly associated with decreased patient survival.^17^ However, until now, the association between *P4HA1* and HNSCC as well as its clinical value was not clearly delineated.

In this study, we evaluated the expression of *P4HA1* in HNSCC and its clinical value. In addition, we also investigated the enrichment of *P4HA1* co‐expressed genes in KEGG pathways to explore its underlying mechanism in HNSCC.

## 2 MATERIALS AND METHODS

### 2.1 Bioinformatic analysis using UCSC Xena browser

*P4HA1* mRNA expression and details of the clinicopathological characteristics of patients with primary HNSCC in TCGA cohort (Project Id: TCGA‐HNSC) were obtained by using the University of California Santa Cruz (UCSC) Xena browser (://xenabrowser.net/).

### 2.2 Comparison of *P4HA1* gene expression between tumor vs non‐tumor samples using Gene Expression Omnibus database

*P4HA1* mRNA expression in HNSCC samples compared with normal tissue was also analyzed using published databases (GSE6631) 18 downloaded from Gene Expression Omnibus (GEO). In the GSE6631 database, data for gene expression profiling of 22 paired HNSCC samples and corresponding adjacent normal tissues were obtained.

### 2.3 Specimens collection

To validate the findings of the bioinformatics analysis, 162 HNSCC tissues and their adjacent non‐tumorous tissues were collected from the Ningbo Medical Centre Lihuili Hospital and the Affiliated Tumor Hospital of Xiangya Medical School, from February 2014 to November 2018. None of the patients underwent treatment before operation. Each specimen was histopathologically confirmed by two pathologists. All specimens were preserved in RNA‐fixer Reagent (Bioteke) and stored at −80°C until further experiments. This study was approved by the Human Research Ethics Committee of Ningbo Medical Centre Lihuili Hospital and the Affiliated Tumor Hospital of Xiangya Medical School. Written informed consent was obtained from all patients.

### 2.4 Total RNA extraction and quantitative real‐time PCR

Total RNA was extracted from 162 paired HNSCC and normal tissues using the TRIzol reagent (Invitrogen), then reverse transcribed into cDNA by GoScript Reverse Transcription (RT) System (Promega) following the manufacturer's instructions. Real‐time quantitative reverse transcription‐polymerase chain reaction quantitative real‐time PCR (qRT‐PCR) was performed as previously described.^19^ The housekeeping gene glyceraldehyde 3‐phosphate dehydrogenase (*GAPDH*) was used as a normalize control. The primers were synthesized by Huada Biotech. The sequences of the PCR primers were as follows: 5′‐AGTACAGCGACAAAAGATCCAG‐3′ and 5′‐CTCCAACTCACTCCACTCAGTA‐3′ for *P4HA1*; 5′‐CCATGGAGAAGGCTGGGG‐3′, and 5′‐CAAAGTTGTCATGGATGACC‐3′ for *GAPDH*. The conditions of thermal cycling were as follows: 95°C at 10 minutes for a hot‐start, 45 amplification cycles at 95°C for 15 seconds, 55°C for 35 seconds, and 70°C for 30 seconds. The expression of *P4HA1* was calculated using the ΔCt method. Larger ΔCt value indicates lower expression. All experiments were performed in triplicate.

### 2.5 Immunohistochemistry staining

P4HA1 protein expression levels in HNSCC tissues and in normal tissues were explored using immunohistochemistry (IHC) staining data from the Human Protein Atlas (HPA; ://www.proteinatlas.org/).

### 2.6 *P4HA1* genetic alteration analysis using cBioPortal for Cancer Genomics and KEGG analysis using ClueGo in Cytoscape

*P4HA1* genetic alterations in HNSCC were examined using cBioPortal for Cancer Genomics (http://www.cbioportal.org/). The associations between *P4HA1* genetic alterations and overall survival (OS) as well as disease‐free survival (DFS) in HNSCC patients were assessed by generating Kaplan‐Meier survival curves. The genes co‐expressed with *P4HA1* in HNSCC were defined as (|Pearson's *r*| ≥ .4 and |Spearman's *r*| ≥ .4). Then, the co‐expressed genes were loaded into ClueGo in Cytoscape for analysis of KEGG pathways. Only pathways with a *P‐*value ≥.05 were included.

### 2.7 Statistical analysis

All statistical analysis was performed using Statistical Program for Social Sciences (SPSS) 20.0 software (SPSS Inc) and R 3.1.2 software (https://www.r-project.org/), which were also used to generate figures. For comparisons of *P4HA1* expression between groups, independent Student's *t* test and one‐way analysis of variance (one‐way ANOVA) tests were employed as appropriate. Receiver operating characteristic (ROC) analysis was used to assess the diagnostic value of *P4HA1* expression for HNSCC. The cut‐off point was defined as the maximum Youden index. HNSCC patients with integrated survival data were divided into high and low *P4HA1* expression groups according to the maximum Youden index based on ROC curves for death and recurrence detection in HNSCC patients. Kaplan‐Meier curves of overall survival (OS) and recurrent‐free survival (RFS) after initial therapy were generated, and log‐rank tests were performed to evaluate the difference between the survival curves. Univariate and multivariate Cox regression analyses were performed to determine the independent prognostic value of *P4HA1* expression in terms of OS and RFS in HNSCC patients. *P*‐value <.05 was considered to be statistically significant.

## 3 RESULTS

### 3.1 *P4HA1* expression is significantly elevated in HNSCC tissues

**Figure 1:**
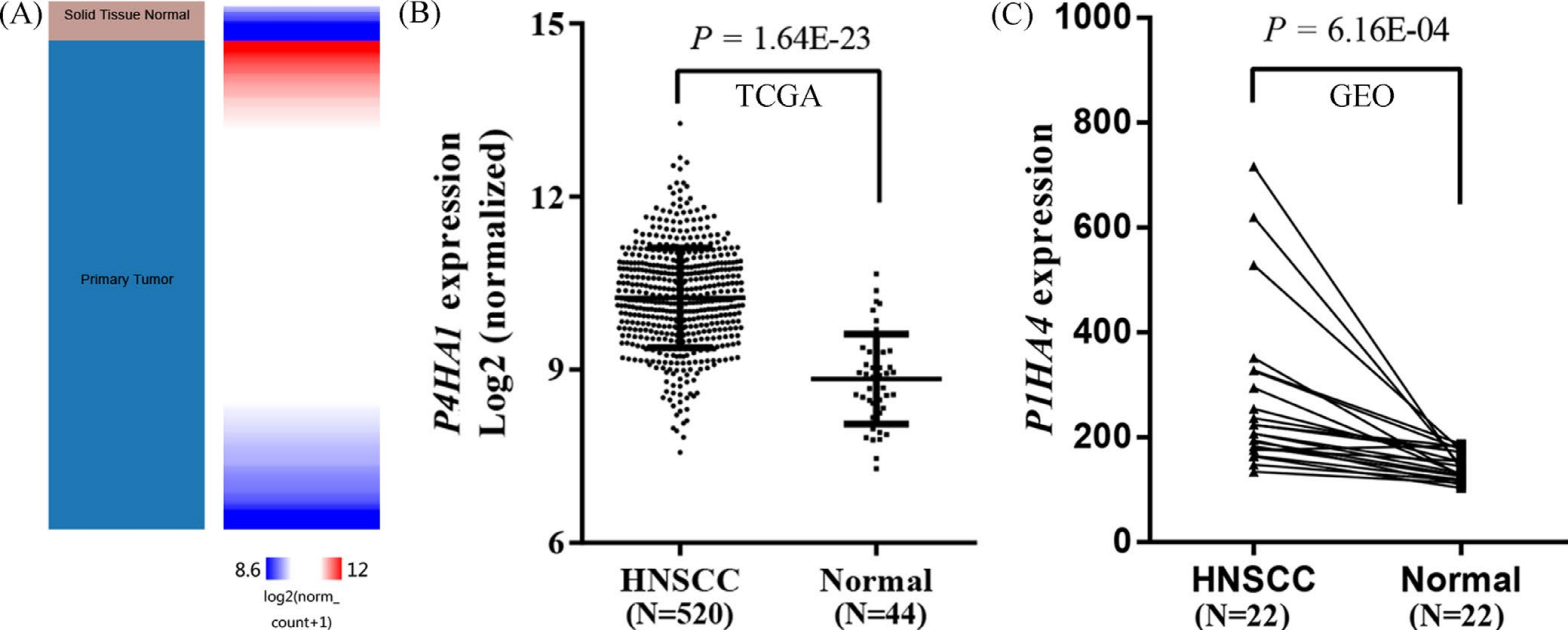
*P4HA1* expression levels are significantly elevated in HNSCC tissues compared with normal tissues using public databases. A–B, Heatmap (A) and plot (B) showing *P4HA1* expression in HNSCC tissue and normal tissue using TCGA database. C, *P4HA1* expression in HNSCC tissue and normal tissue using GEO database. N, Sample number

**Figure 2:**
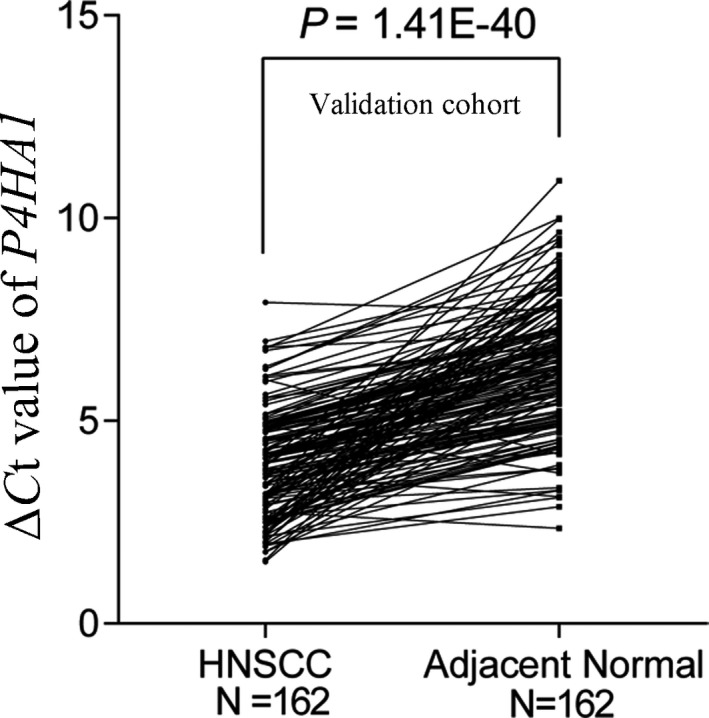
*P4HA1* expression levels were significantly higher in HNSCC tissues vs paired non‐tumor tissues in our validation cohort. N, sample number

**Figure 3:**
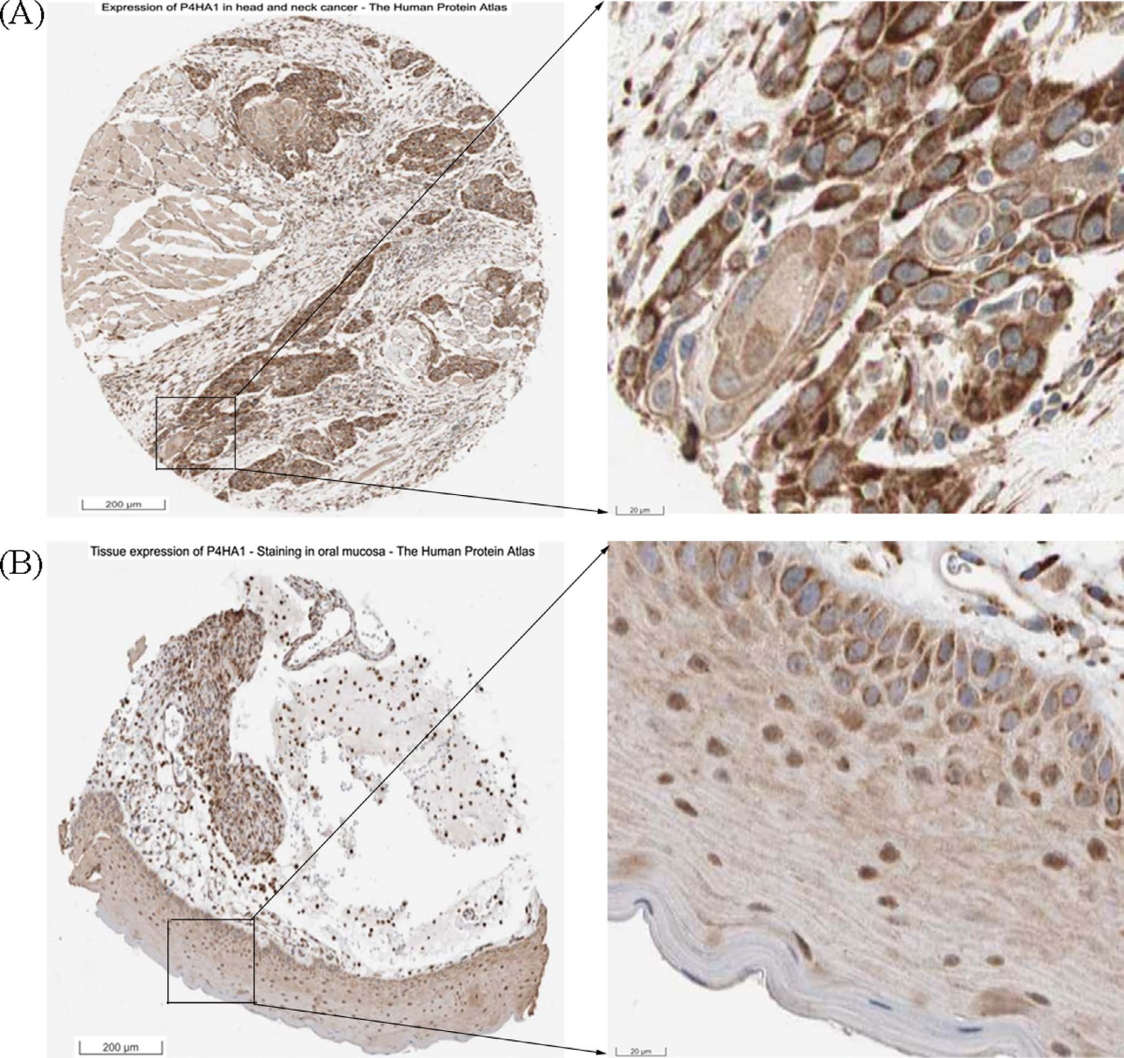
P4HA1 immumohistochemical staining images in HNSCC tissue and normal tissue. Images were obtained from Human Protein Atlas (://v18.proteinatlas.org/). A, High P4HA1 protein expression in HNSCC tissue (://www.proteinatlas.org/ENSG00000122884-P4HA1/pathology/tissue/head+and+neck+cancer#img). B, Medium P4HA1 protein expression in normal oral mucosa (https://www.proteinatlas.org/ENSG00000122884-P4HA1/tissue/oral+mucosa#img)

By comparing *P4HA1* mRNA expression using the RNA‐Seq data of 520 HNSCC tissues and normal tissues in TCGA, we revealed that HNSCC tissues exhibited significantly elevated *P4HA1* mRNA expression compared with normal tissues (*P* = 1.64E‐23; Figure 1A,B), consistent with our findings using GEO data (*P* = 6.16E‐04; Figure 1C). The qRT‐PCR analysis using the 162 paired HNSCC samples confirmed that *P4HA1* mRNA expression levels were significantly upregulated in HNSCC tissues compared with adjacent normal tissues (*P* = 1.41E‐40, Figure 2). We further explored P4HA1 protein expression in HNSCC tissues and normal tissues using the HPA database. Immunohistochemical staining images revealed that P4HA1 exhibited high expression in HNSCC tissues (Figure 3A). In comparison, oral mucosa exhibited medium P4HA1 expression (Figure 3B).

### 3.2 Association of *P4HA1* expression with some clinical features of HNSCC

Then, we analyzed the association between *P4HA1* mRNA expression levels and clinicopathological characteristics of patients with HNSCC. As shown in Table 1, high *P4HA1* expression in HNSCC tissues was significantly associated with alcohol consumption (*P* = .019), tumor location (*P* = .017), HPV infection (*P* = .011), tumor category (*P* = .006), lymphatic metastasis (*P* = .006), and pathological stage (*P* = .002).

**Table 1:** Association between *P4HA1* expression and clinicopathological features of HNSCC patients

Characteristics	N	Mean ± SD	*P*‐value
Gender			
Female	136	10.168 ± 0.877	0.208
Male	384	10.276 ± 0.858	
Age			
<60 y	233	10.229 ± 0.827	0.63
≥60 y	286	10.265 ± 0.894	
Smoking history			
No	117	10.149 ± 0.894	0.199
Yes	391	10.266 ± 0.853	
Alcohol history			
No	162	10.115 ± 0.892	0.019
Yes	347	10.306 ± 0.842	
Histologic grade			
G1 + 2	366	10.199 ± 0.830	0.084
G3 + 4	132	10.350 ± 0.927	
Tumor site			
Oral cavity + oropharynx	394	10.197 ± 0.870	0.017
Hypopharynx + larynx	126	10.408 ± 0.824	
HPV status			
Negative	73	10.328 ± 0.724	0.011
Positive	38	9.942 ± 0.798	
Tumor category			
Tis/T1/T2	185	10.104 ± 0.853	0.006
T3/T4	273	10.330 ± 0.850	
Lymphatic metastasis			
No	176	10.111 ± 0.820	0.006
Yes	244	10.341 ± 0.862	
Pathological stage			
I + II	101	10.021 ± 0.812	0.002
III + IV	347	10.329 ± 0.875	

Abbreviation: N, sample number.

### 3.3 Diagnostic value of *P4HA1* expression for HNSCC

**Figure 4:**
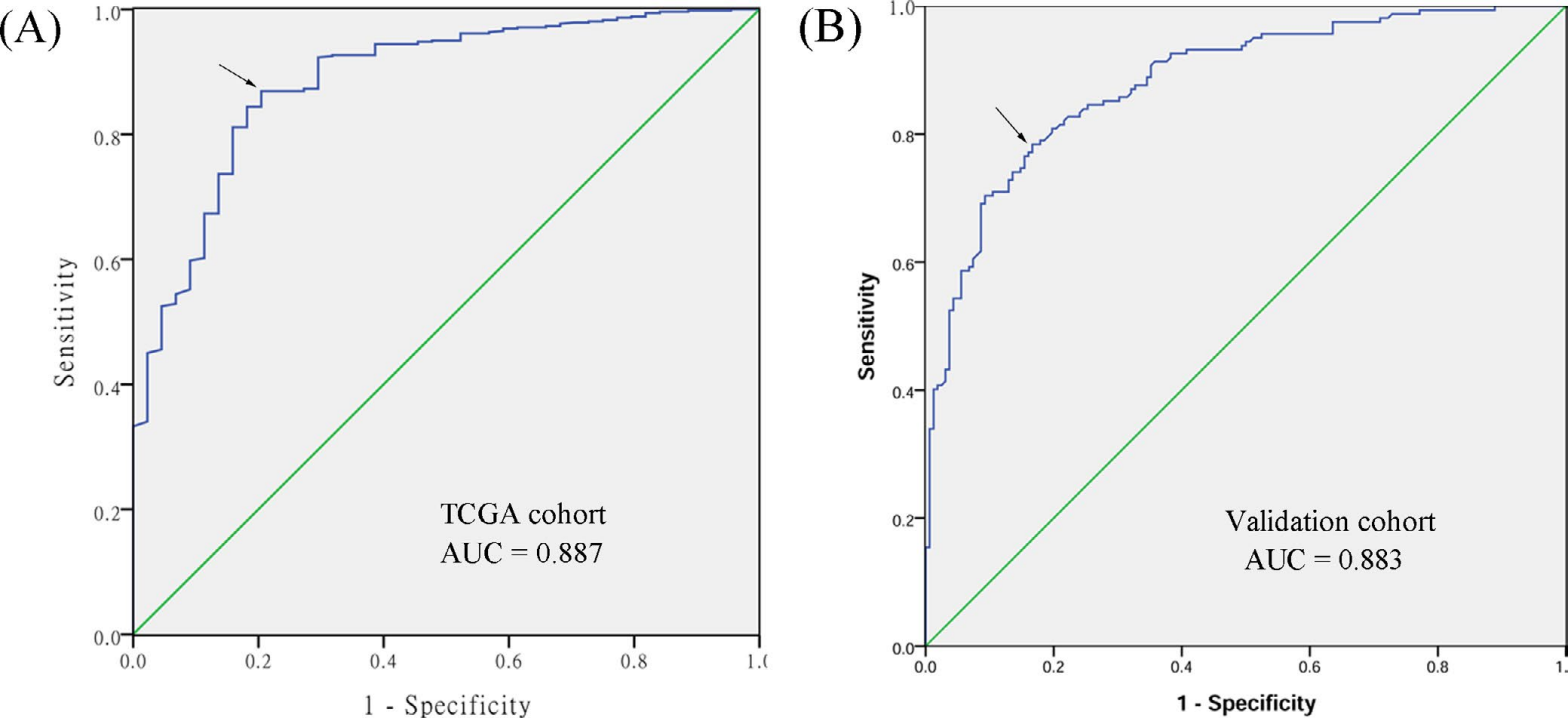
Receiver operating characteristic (ROC) curves to assess the diagnostic value of *P4HA1* expression in HNSCC patients. The area under the curve (AUC) was 0.887 based on TCGA cohort. The area under the curve (AUC) was 0.883 based on our validation cohort. The arrow points to the intercept

We examined the diagnostic value of *P4HA1* expression in HNSCC using ROC curves. An area under the ROC curve (AUC) closer to 1.0 signifies that the test exhibits more perfect discrimination. The maximum Youden index was used as a cut‐off point. The result suggested that *P4HA1* expression yielded an AUC of 0.887, a sensitivity of 88.8%, and a specificity of 78.1% using TCGA cohort (Figure 4A) and yielded an AUC of 0.883, a sensitivity of 78.4% and a specificity of 83.3% using our validation cohort (Figure 4B).

### 3.4 High *P4HA1* expression was an independent prognostic predictor of unfavorable OS and RFS in HNSCC patients

Using the maximum Youden index as cut‐off point (10.665), we classified 517 HNSCC patients with integrated OS data into the high *P4HA1* expression group (N = 173) and low *P4HA1* expression group (N = 344). Kaplan‐Meier curves and log‐rank tests revealed that high *P4HA1* expression was associated with significantly worse OS in HNSCC (*P* = 2.07E‐5). In addition, 437 HNSCC patients with integrated RFS data were divided into high (N = 106) and low (N = 331) *P4HA1* expression groups according to a cut‐off value of 10.845. Kaplan‐Meier curves and log‐rank tests revealed that HNSCC patients in the high *P4HA1* expression group exhibited significantly poorer RFS (*P* = .002).

**Figure 5:**
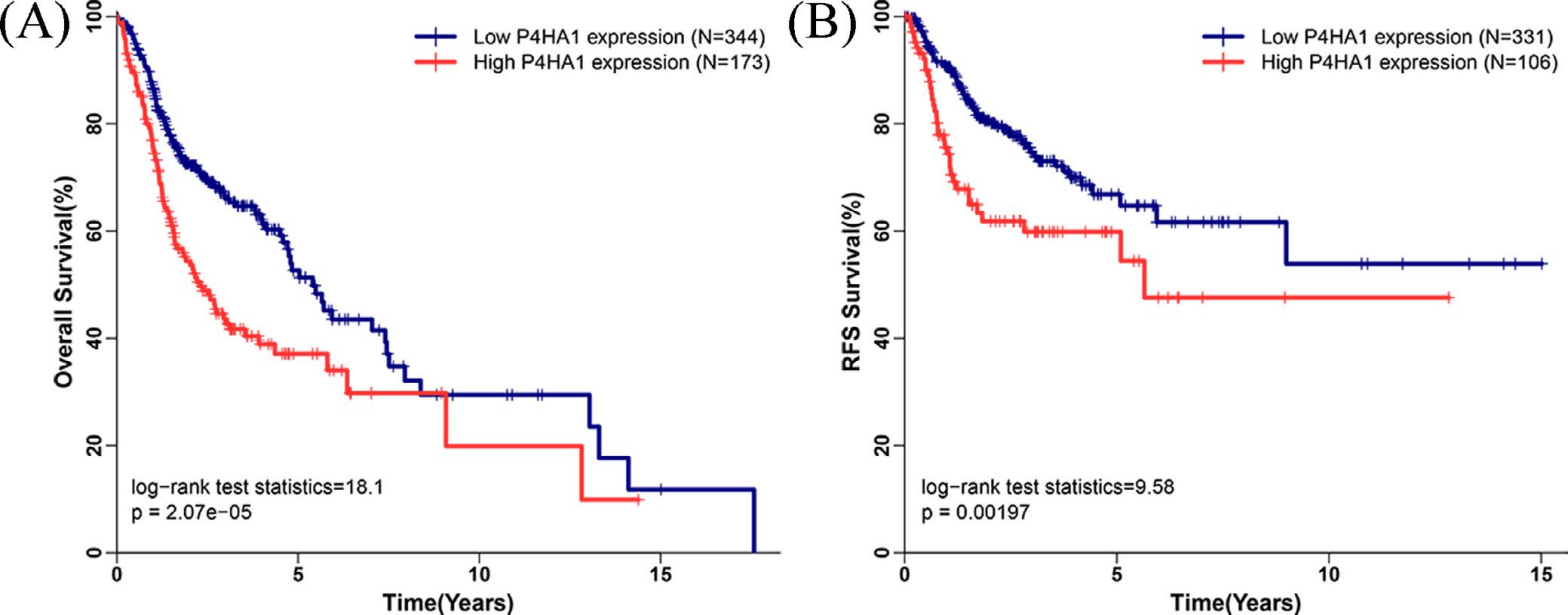
Association between *P4HA1* expression and survival in HNSCC. A, High *P4HA1* expression is associated with poor OS in HNSCC patients; B, High *P4HA1* expression is associated with poor RFS in HNSCC patients

In univariate Cox proportional hazards analysis, the results showed that elderly (hazard ratio (HR): 1.318, 95% confidence interval (CI): 1.003‐1.731, *P* = .047), female (HR: 1.349, 95% CI: 1.014‐1.796, *P* = .04), advanced stages (HR: 1.754, 95% CI: 1.203‐2.558, *P* = .004), lymphatic metastasis (HR: 1.86, 95% CI: 1.343‐2.576, *P* = 1.86E‐04), and elevated *P4HA1* expression (HR: 1.775, 95% CI: 1.358‐2.321, *P* = 2.68E‐05) were associated with unfavorable OS. Of note, we found that alcohol consumption (HR: 1.809, 95% CI: 1.130‐2.896, *P* = .014), advanced stages (HR: 2.302, 95% CI: 1.249‐4.242, *P* = .007), lymphatic metastasis (HR: 1.653, 95% CI: 1.062‐2.573, *P* = .026), and high *P4HA1* expression (HR: 1.865, 95% CI: 1.249‐2.785, *P* = .002) were significantly associated with shorter RFS (Table 2). Multivariate Cox proportional hazard analysis was conducted to investigate the independent prognostic factors in terms of OS and RFS in HNSCC patients by adjusting only variables that exhibited significance in univariate analysis. We found that high *P4HA1* expression (OS: HR: 1.728, 95% CI: 1.267‐2.357, *P* = .001; RFS: HR: 2.025, 95% CI: 1.296‐3.162, *P* = .002) was independent unfavorable prognostic factor in terms of OS and RFS in HNSCC patients (Figure 5).

**Table 2:** Univariate and multivariate analysis of overall survival and recurrent‐free survival in HNSCC patients

Characteristics	Univariate analysis			Multivariate analysis		
	HR	95% CI	*P* value	HR	95% CI	*P* value
Overall survival						
Age (≥60 y vs <60 y)	1.318	1.003‐1.731	.047	1.223	0.891‐1.679	.214
Gender (female vs male)	1.349	1.014‐1.796	.04	1.372	0.978‐1.926	.067
Smoking history (yes vs no)	1.123	0.803‐1.572	.498			
Alcohol history (yes vs no)	0.942	0.709‐1.252	.68			
Histologic grade (G3/4 vs G1/2)	0.867	0.637‐1.180	.419			
Pathologic stage (III/IV vs I/II)	1.754	1.203‐2.558	.004	1.878	1.055‐3.345	.032
Pathologic N (N1/2/3 vs N0)	1.86	1.343‐2.576	1.86E‐04	1.422	0.973‐2.078	.069
HPV (positive vs negative)	0.856	0.420‐1.746	.67			
*P4HA1* expression (high vs low)	1.775	1.358‐2.321	2.68E‐05	1.728	1.267‐2.357	.001
Recurrence‐free survival						
Age (≥60 y vs <60 y)	1.291	0.878‐1.899	.194			
Gender (female vs male)	1.118	0.714‐1.751	.626			
Smoking history (yes vs no)	0.973	0.626‐1.513	.904			
Alcohol history (yes vs no)	1.809	1.130‐2.896	.014	1.36	0.827‐2.236	.226
Histologic grade (G3/4 vs G1/2)	0.821	0.526‐1.281	.384			
Pathologic stage (III/IV vs I/II)	2.302	1.249‐4.242	.007	1.514	0.721‐3.179	.274
Pathologic N (N1/2/3 vs N0)	1.653	1.062‐2.573	.026	1.19	0.709‐1.996	.511
HPV (positive vs negative)	0.914	0.343‐2.438	.858			
*P4HA1* expression (high vs low)	1.865	1.249‐2.785	.002	2.025	1.296‐3.162	.002

Abbreviations: CI, confidence interval; HR, hazard ratio.

**Figure 6:**
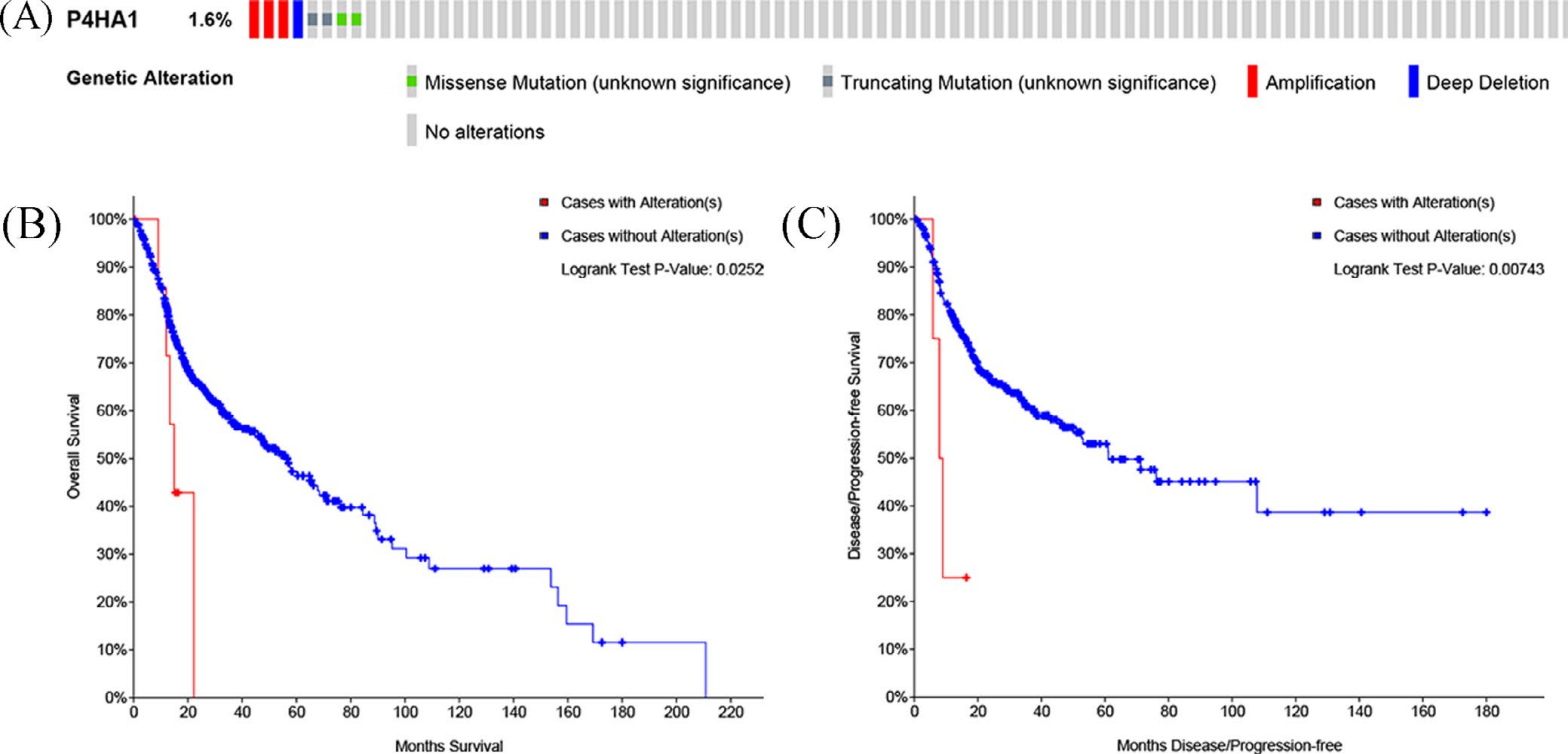
*P4HA1* genetic alterations in HNSCC and its correlation with prognosis of HNSCC patients in OS and DFS. A, *P4HA1* is altered in 1.6% (8/504) of sequenced HNSCC patients. B, *P4HA1* genetic alterations were associated with significantly worse overall survival; C, *P4HA1* genetic alterations were associated with significantly worse disease‐free survival

**Figure 7:**
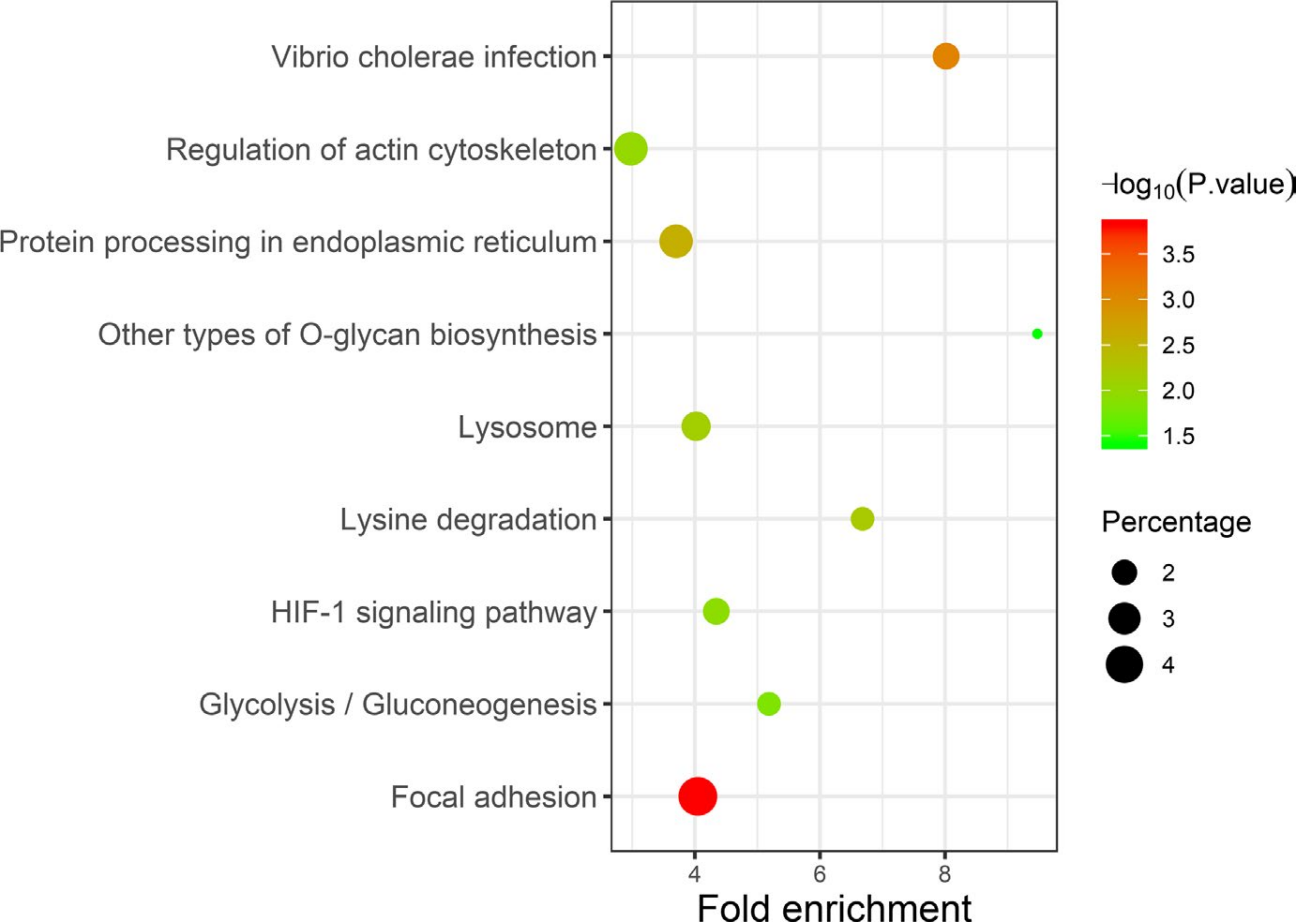
KEGG pathway analysis of the genes co‐expressed with *P4HA1* in HNSCC

### 3.5 *P4HA1* genetic alteration was associated with worse OS and DFS in HNSCC patients

The cBioPortal for Cancer Genomics was utilized to explore *P4HA1* genetic alterations in HNSCC *P4HA1* was only altered in 8 samples, including 504 sequenced HNSCC patients from the Cancer Genome Atlas Research (Figure 6). Then, we also evaluated the association between *P4HA1* genetic alteration and survival in HNSCC patients. Survival curves indicated that HNSCC patients with *P4HA1* genetic alterations exhibited significantly worse OS (log‐rank *P* = .025) and DFS (log‐rank *P* = .007).

### 3.6 KEGG analysis based on *P4HA1* co‐expressed genes

By data mining using cBioPortal for Cancer Genomics, we identified 282 co‐expressed genes with *P4HA1* in HNSCC. To further investigate the possible signaling pathways that *P4HA1* might be involved in, *P4HA1* co‐expressed genes in HNSCC were subjected to KEGG pathway analysis. In HNSCC, *P4HA1* co‐expressed genes were enriched in the HIF‐1 signaling pathway, focal adhesion, regulation of actin cytoskeleton, protein processing in endoplasmic reticulum, vibrio cholera infection, lysosome, lysine degradation, glycolysis/gluconeogenesis, and other types of O‐glycan biosynthesis (Figure7).

## 4 DISCUSSION

Collagens are the major structural extracellular matrix (ECM) proteins and form fibers or networks in tumor tissues to support the tumor microenvironment and play crucial roles in carcinogenesis.^20^, ^21^ *P4HA1* is a key intracellular enzyme to catalyze the formation of 4‐hydroxyproline that is essential for proper three‐dimensional folding of newly synthesized procollagen chains, maintaining ECM homeostasis.^14^ Accumulating evidence indicates that increased *P4HA1* is associated with the initiation, invasion, and metastasis of many human cancers, including hepatocellular carcinoma,^22^ breast cancer,^17^ and prostate cancer.^23^ However, the association of *P4HA1* with HNSCC remains uninvestigated. In the present study, significantly increased *P4HA1* mRNA levels were observed in HNSCC tissues compared with nontumor tissues using TCGA database, which is consistent with the analysis results of the GEO database. Furthermore, the HPA database validated that P4HA1 protein levels were elevated in HNSCC compared with surrounding normal tissue and demonstrated that P4HA1 was mainly localized to the endoplasmic reticulum and slightly localized to the mitochondria and vesicles. All these results suggested that *P4HA1* plays an important role in HNSCC transformation.

The history of high alcohol consumption is a crucial factor for increased the risk of HNSCC.^24^ In this study, using RNA‐seq data in TCGA‐HNSC, we found that increased *P4HA1* expression was significantly correlated with alcohol consumption, suggesting that alcohol might contribute to HNSCC by inducing *P4HA1* expression. Accumulating evidence indicates that HPV infection is an important risk factor for HNSCC. HPV‐positive HNSCCs and HPV‐negative HNSCCs differ with respect to the molecular mechanisms underlying their oncogenic processes.^4^ HPV‐positive cancers are more susceptible to chemotherapy and radiation with better prognosis compared with HPV‐negative patients.^25^, ^26^ Studies have consistently demonstrated that most HNSCCs with HPV detected in the tumor are from the oral cavity and oropharynx,^27^ and HPV is driving the increasing incidence of oral cavity and oropharyngeal cancer over the past 30 years.^28^, ^29^ Consistent with prior reports that integration of HPV into the genome results in altered DNA copy number and mRNA transcript abundance and splicing,^30^ our analysis demonstrated that downregulated *P4HA1* was more frequently found in tumors located in the oral cavity and oropharynx as well as in HPV‐positive patients, indicating that HPV infection contributes to HNSCC by inhibiting *P4HA1* expression. Moreover, *P4HA1* was overexpressed in HNSCC at advanced stages and with lymphatic metastasis compared with early stage disease and no lymphatic metastasis, suggesting the involvement of *P4HA1* in the tumorigenesis and metastatic progression of HNSCC.

One of the most important issues concerning cancer patients is how to screen and diagnose at an early stage. Screening for HNSCC depends on clinical symptoms and imaging examinations (laryngoscopy, computed tomography, magnetic resonance imaging, and positron emission tomography), and a definite diagnosis depends on biopsy and histopathological examination.^1^ However, given the nonspecificity of symptoms in the early stage, the early detection of HNSCC remains unsatisfactory. In the present study, we constructed ROC curves and calculated the AUC to determine the diagnostic value of *P4HA1* for HNSCC. The AUC value of TCGA and validation cohort was 0.887 and 0.883, respectively, signifying greater diagnostic accuracy compared with conventional cancer‐related biomarkers, such as carcinoembryonic antigen (CEA), squamous cell carcinoma antigen (SCC Ag), TPS (tissue polypeptide specific antigen), and Cyfra 21‐1.^31^, ^32^ These results suggest that *P4HA1* expression levels might represent a promising diagnostic biomarker for HNSCC.

Despite current treatment regimens with curative intent, including surgery, radiotherapy and chemotherapy, local or distant recurrence rates remain high, and the 5‐year overall survival rate of HNSCC patients is less than 50%.^33^ Emerging therapeutic strategies, such as anti‐EGFR antibody (cetuximab) and anti‐PD‐1 antibodies (pembrolizumab and nivolumab) that have recently been approved for the treatment of advanced and metastatic HNSCC, are promising options for the management of high‐risk patients. However, predicting high‐risk HNSCC patients remains a challenge for both the clinician and the pathologist.^34^ Tumor diameter, lymphatic metastasis, distal metastasis, and clinical stage are vital factors affecting tumor patient outcomes;^35^ however, these factors are unable to absolutely justify clinical application due to heterogeneous molecular mechanisms and clinical behaviors of HNSCC.^36^ Therefore, reliable prognostic biomarkers are urgently needed to identify HNSCC patients at risk of disease recurrence and subsequent death. Recently, dysregulation of *P4HA1* expression was reported to promote tumor progression and associated with unfavorable prognosis in various cancers, including gliomas,^16^ breast cancer,^17^ and prostate cancer.^23^ With regard to the findings in the present study, the log‐rank test and univariate Cox proportional hazard analysis showed that high *P4HA1* expression was correlated with inferior OS and RFS of HNSCC patients, and these findings are consistent with previous report.^37^ Future multivariate Cox proportional hazard analysis confirmed that both elevated *P4HA1* and advanced stages were dependent poor prognostic factors for OS and RFS of HNSCC patients after adjusting for age, gender, smoking behavior, alcohol consumption, and histologic grade. Taken together, the present study indicates that *P4HA1* expression may be of great value for tailoring of individual therapies and risk stratification of recurrence and subsequent death, which might help these patients benefit from an intensified first‐line treatment and surveillance.

Based on large HNSCC samples in TCGA using cBioPortal for Cancer Genomics, we found that although *P4HA1* genetic alteration was less frequent in HNSCC (8/504), its alteration was associated with significantly worse overall survival and disease‐free survival. Given that elevated *P4HA1* was dependent poor prognostic biomarker for OS and RFS of HNSCC patients, we hypothesized that *P4HA1* genetic alterations might increase its expression level, which should be confirmed in further investigations. In HNSCC, *P4HA1* coexpressed genes were additionally enriched in some cancer‐related and metabolism‐related pathways, such as HIF‐1 signaling pathway, lysine degradation pathway, and gluconeogenesis pathway. These results can provide novel insight HNSCC pathogenesis. In breast cancer, HIF‐1 mediates increasing *P4HA1* expression in conditions of hypoxic stress, resulting in fibrillary collagen deposition and the induction of a more invasive cell phenotype.^17^ However, several limitations of our study should be considered. Due to the size of sample, we did not validate the P4HA1 protein expression level in HNSCC tissues. Additionally, the role of *P4HA1* in these pathways in the HNSCC is not completely clear. Thus, further studies are needed to explore underlying mechanism of *P4HA1* in these pathways in the HNSCC.

## 5 CONCLUSIONS

This integrated bioinformatics analysis provides strong evidence that increasing *P4HA1* is significantly associated with HNSCC carcinogenesis and metastasis. Additionally, high *P4HA1* expression is both a diagnostic biomarker and an independent prognostic factor for poor OS and RFS in HNSCC patients.

## CONFLICTS OF INTEREST

None of the authors have any commercial or other associations that might pose a conflict of interest.
